# Allocation of resources in working memory: Theoretical and empirical implications for visual search

**DOI:** 10.3758/s13423-021-01881-5

**Published:** 2021-03-17

**Authors:** Stanislas Huynh Cong, Dirk Kerzel

**Affiliations:** Faculty of Psychology and Educational Sciences, University of Geneva, 40 Boulevard du Pont d’Arve, 1205 Geneva, Switzerland

**Keywords:** Visual search, Top-down control, Attentional template, Working memory, Resource allocation

## Abstract

Recently, working memory (WM) has been conceptualized as a limited resource, distributed flexibly and strategically between an unlimited number of representations. In addition to improving the precision of representations in WM, the allocation of resources may also shape how these representations act as attentional templates to guide visual search. Here, we reviewed recent evidence in favor of this assumption and proposed three main principles that govern the relationship between WM resources and template-guided visual search. First, the allocation of resources to an attentional template has an effect on visual search, as it may improve the guidance of visual attention, facilitate target recognition, and/or protect the attentional template against interference. Second, the allocation of the largest amount of resources to a representation in WM is not sufficient to give this representation the status of attentional template and thus, the ability to guide visual search. Third, the representation obtaining the status of attentional template, whether at encoding or during maintenance, receives an amount of WM resources proportional to its relevance for visual search. Thus defined, the resource hypothesis of visual search constitutes a parsimonious and powerful framework, which provides new perspectives on previous debates and complements existing models of template-guided visual search.

## Introduction

We spend a large part of our daily lives searching for known objects in dense visual scenes, such as car keys on a cluttered desk or a child’s jacket in a crowded playground. That is, our visual environment comprises an overwhelming amount of information from which we must select a limited quantity that is of interest. To achieve this complex operation, an accurate representation of the relevant features, for instance the shape of the car keys or the color of the child’s jacket, may give a significant advantage in achieving efficient visual search. This theoretical review examines how these two abilities – looking for goal-relevant objects and remembering their features – interact in a way that optimizes behavior.

Working memory (WM) commonly refers to the processes that maintain and manipulate representations most needed for ongoing cognitive operations (Baddeley, 2010; Cowan, 2017; Oberauer, 2019). WM involves a broad network of brain areas (Christophel et al., 2017; D'Esposito & Postle, 2015; Postle, 2006) and is considered a core cognitive ability sustaining a large range of processes, from perception to problem solving and fluid intelligence (Engle, 2002, 2018; Miller & Cohen, 2001; Unsworth et al., 2014). In one of the earliest conceptualization of WM, Baddeley and Hitch (1974) described a system in which *internal attention* (see Table 1) regulates WM and coordinates activity between its components. In this multicomponent model, WM was dedicated to the short-term maintenance and processing of information, involving limited domain-specific stores and an executive attention system. Subsequent state-based models of WM (Cowan, 1999, 2005; Gilchrist & Cowan, 2011; McElree, 2001, 2006; Oberauer, 2002, 2009; Oberauer & Hein, 2012) challenged the idea of multiple components and proposed that WM comprises a handful of representations activated from long-term memory (LTM) by the *focus of attention.* In a similar vein, controlled-attention models (Engle, 2002; Kane et al., 2001) relied on investigations of individual differences to posit that WM was the general attention capacity for maintaining a restricted amount of active information and protecting it from interference or time-based decay (Barrouillet et al., 2004; Barrouillet & Camos, 2007). While these major models of WM differ substantially, they share at least two common assumptions. First, internal attention plays a critical role in controlling the activation, maintenance, and processing of WM representations, corroborating the idea that WM and internal attention are intimately linked (Awh et al., 2006; Chun, 2011; Gazzaley & Nobre, 2012; Kiyonaga & Egner, 2013; Myers et al., 2017; Oberauer, 2019; Souza & Oberauer, 2016). Second, WM is extremely limited in capacity with estimates pointing towards a maximum of approximately four representations, whether verbal (Cowan, 2001) or visual (Vogel et al., 2001). In that sense, WM capacity has been classically defined as the number of remembered items using discrete or categorical stimulus sets, such as letters, digits, or easily identifiable colors. However, over the past two decades, the explosion of research using more precisely controlled visual paradigms (see Schurgin, 2018) led to redefining the capacity of WM and to conceptualizing the role of internal attention in more detail. In particular, internal attention may optimize the limited storage space in WM by prioritizing behaviorally relevant over irrelevant information. Serving this function, internal attention is thought to act both as a “filter” that determines what information gains access to WM (Awh & Vogel, 2008) and as a “resource” that is flexibly allocated amongst stored representations based on their respective relevance (Franconeri et al., 2013; Ma et al., 2014).

**Table 1:** Glossary

**Internal attention:** Attentional selection and enhancement of internal information that is currently not available to the senses, such as WM or LTM representations, intended actions and goals, tasks rules and corresponding responses.	
**Focus of attention:** Mechanism of internal attentional selection that grants access to a specific state in WM. Depending on the model, only one or several representations can access this specific WM state to be updated, manipulated, and recalled.	
**Contralateral delay activity (CDA):** Sustained negativity recorded at posterior cortical locations (e.g., parietal) contralateral to the stimuli being maintained in WM.	
**Visual attention:** Attentional selection and enhancement of visual information that is currently present in the environment, such as basic features (e.g., colors, orientations, or shapes), entire objects, and specific locations.	
**Posterior alpha-band oscillations**: Structured rhythmicity in the frequency range of 8–14 Hz that is recorded at posterior cortical locations (e.g., parietal). Attenuation (vs. amplification) of alpha oscillations is generally associated with increased (vs. decreased) cortical engagement.	
**Retro-cue:** Spatial cue presented during the retention interval to guide internal attention toward a subset of WM representations that are most relevant.	

### Attentional filter and WM resources

According to filter models, internal attention serves as a gatekeeper that controls the flow of information into WM so that only the most relevant representations consume the limited storage space. That is, the attentional filter selects appropriate information for encoding in WM (Gazzaley, 2011; Murray et al., 2011; Schmidt et al., 2002) and prevents distracting information from gaining access to it (Awh & Vogel, 2008; Cowan & Morey, 2006; Cusack et al., 2009; Gazzaley, 2011; McNab & Klingberg, 2008; Vissers et al., 2016; Vogel et al., 2005; Zanto & Gazzaley, 2009). In this view, individual differences in WM capacity are determined by the efficiency of the attentional filter, rather than by differences in the storage space per se. To examine the proportion of relevant and irrelevant information entering WM, the *contralateral delay activity* (CDA), an electrophysiological correlate of the number of representations maintained in WM (Luria et al., 2016; Vogel & Machizawa, 2004; Vogel et al., 2005), has been recorded in two types of individuals. Particularly, high-capacity individuals were shown to selectively encode relevant representations (i.e., only targets), whereas low-capacity individuals stored additional irrelevant representations (i.e., both targets and non-targets) as evidenced by systematically larger CDAs for the latter (Jost & Mayr, 2016; Lee et al., 2010; Liesefeld et al., 2013; Qi et al., 2014; Vogel et al., 2005). Thus, as a result of inefficient attentional filtering, low-capacity individuals may hold a larger number of representations in WM than high-capacity individuals, but these may simply be unnecessary for the task at hand. In these models, internal attention serves as a simple “in or out” filter that determines the proportion of relevant and irrelevant representations entering WM. However, no further control over how these representations are encoded and maintained is considered. That is, an additional mechanism involving internal attention may be necessary to set the goal-relevance of representations, once access to WM is granted.

In contrast to traditional discrete-capacity models (Luck & Vogel, 1997, 2013; Zhang & Luck, 2008), resource models recently proposed that WM relied on a limited attentional resource, distributed flexibly and strategically between an unlimited number of representations (Alvarez & Cavanagh, 2004; Fougnie et al., 2012; Franconeri et al., 2013; Keshvari et al., 2013; Ma et al., 2014; Wilken & Ma, 2004). Specifically, internal representations of sensory stimuli are considered as intrinsically noisy, that is, contaminated by random fluctuations. Depending on the goal-relevance of these stimuli, resources are allocated to reduce the noise in their WM representations, enhancing their precision. However, as resources are limited, the noise level increases with the number of WM representations maintained simultaneously. Consistently, the recall precision declines gradually and continuously with the number of representations in WM, following a power-law function (Bays et al., 2009; van den Berg et al., 2012).^1^ Moreover, the goal-relevance of a stimulus enhances its recall precision (Dube & Al-Aidroos, 2019; Dube et al., 2017; Emrich et al., 2017; Salahub et al., 2019; Zokaei et al., 2011) at the expense of other stimuli (Bays et al., 2011; Bays & Husain, 2008; Gorgoraptis et al., 2011). Thus, like filter models that emphasize the ratio of relevant and irrelevant information accessing WM, resource models do not consider the number of remembered items to be the key measure of WM capacity. Instead, the precision of recall is assumed to directly reflect the allocation of WM resources between stored representations.

### Aim of the review

Here, we focus on the optimization of the limited storage space in WM through the distribution of resources, rather than through attentional filtering. Particularly, we review empirical evidence that the allocation of resources in WM has consequences not only on memory, but also on the exploration of visual environments. In addition to determining the recall precision of representations, we propose that WM resources play a significant role in shaping how these representations interact with visual search. In the first section, we give a concise overview of the recent research on the relationship between WM and visual search. In the second section, we present a theoretical proposal on the role of WM resources in this relationship and assess its empirical plausibility. In the third section, we address three main hypotheses about the functional value of WM resources in visual search. Finally, we conclude on the questions that should be answered with priority in future research aiming at developing the resource hypothesis of visual search.

## WM and visual search

Visual search designates the common task of looking for a particular target object that appears among multiple non-targets at an unpredictable location in the visual field. When one or several visual features of the target object are known in advance, the search process can be enhanced by this knowledge. Consistently, most models of visual search (Bundesen, 1990; Bundesen et al., 2005; Desimone & Duncan, 1995; Huang & Pashler, 2007; Logan, 2002; Schneider, 2013; Wolfe, 1994, 2007, 2020) include the concept of attentional template (Duncan & Humphreys, 1989), attentional control set (Folk et al., 1992), or target template (Vickery et al., 2005). Specifically, attentional templates refer to internal representations of target features that are maintained in WM or LTM during visual search (Carlisle et al., 2011; Woodman & Arita, 2011; Woodman et al., 2013). Activated shortly before the search task (Grubert & Eimer, 2018, 2020), attentional templates selectively prioritize sensory information to locate objects with corresponding attributes (Eimer, 2014) and to determine target-matches (Cunningham & Wolfe, 2014). That is, attentional templates contribute to the guidance of *visual attention* toward potential targets and to the decision about their relevance for current behavior. Although a growing number of studies have investigated template-guided visual search, many questions remain open regarding the status of the attentional template in WM and the number of concurrently active attentional templates. On these issues, two lines of research arrive at different conclusions.

**Fig. 1:**
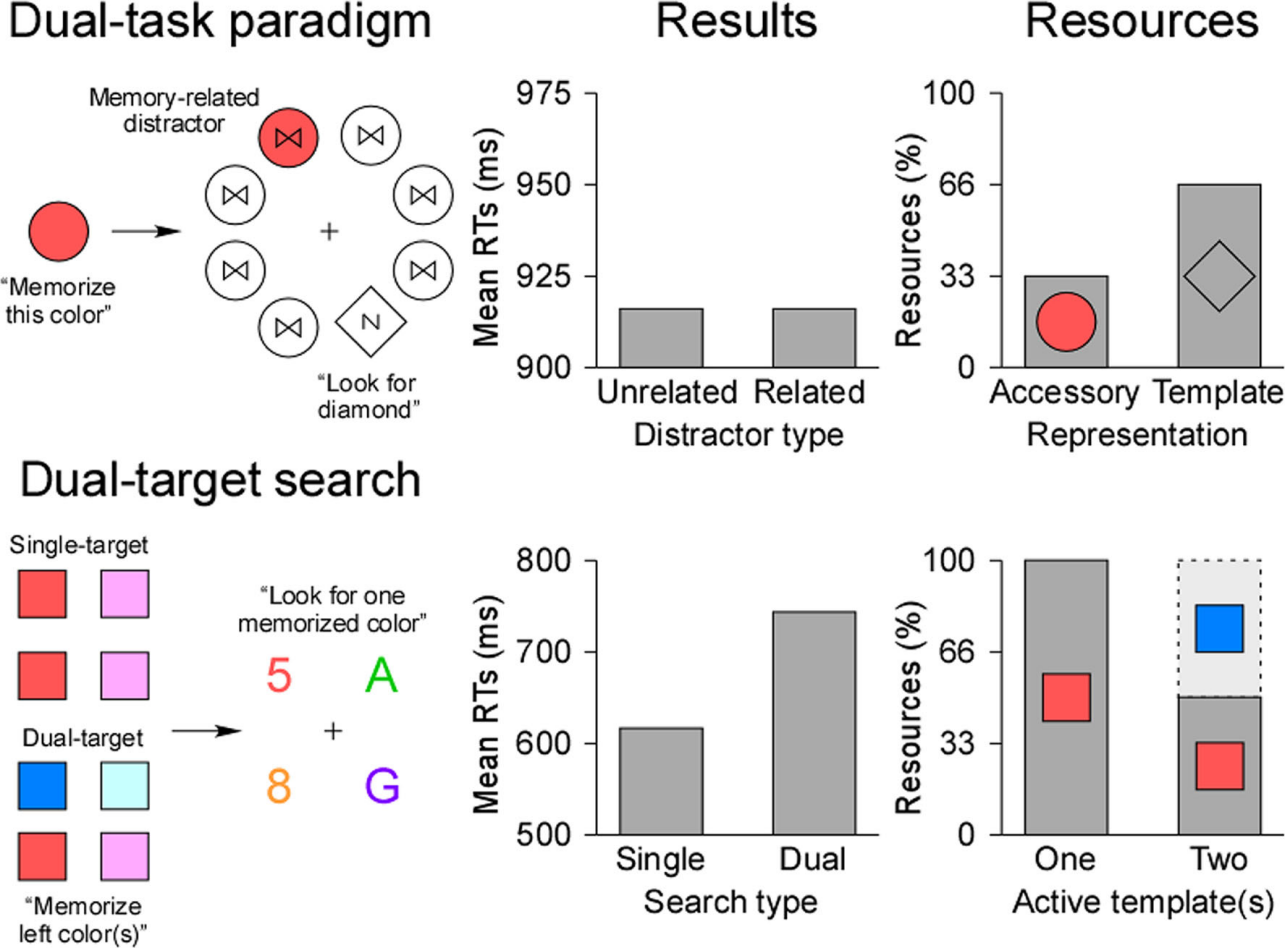
Experimental procedures in the dual-task paradigm and in dual-target search, typical results, and the hypothetical allocation of working memory (WM) resources. The upper left panel depicts an example trial inspired by Olivers (2009) in which observers were asked to memorize a color and then search for a variable shape target. Whether the distractor was in the memorized color or in an unrelated color, mean reaction times (RTs) were similar, indicating the absence of memory-based interference (data from their Experiment 5). More WM resources may be allocated to the attentional template than to the “accessory” color in this case. The lower left panel represents an example trial inspired by Grubert et al. (2016) in which observers had to memorize one or two colors and then search for an alphanumeric character defined by one of these two colors. Mean RTs were delayed in dual- compared with single-target search, suggesting the presence of a cost with multiple attentional templates (data from their Experiment 1). In this situation, a single attentional template may receive all resources, while two active attentional templates may receive an equal share of WM resources

Inspired by state-based models of WM (McElree, 2001, 2006; Oberauer, 2002, 2009; Oberauer & Hein, 2012), the single-template hypothesis (Olivers et al., 2011) proposes a fundamental division in WM between two representational states. In this view, only a single representation may be maintained in an “active” state by the focus of attention, allowing it to serve as an attentional template. In contrast, other representations in WM may be encoded in an “accessory” state, in which they cannot interact with visual search until they become relevant. The implementation and switch between these two states in WM is thought to be reflected in *posterior alpha-band oscillations* (for reviews, see de Vries et al., 2020; van Ede, 2018). The central line of evidence supporting the proposal of Olivers et al. (2011) comes from attentional capture effects (Theeuwes, 1991, 1992) in dual-task paradigms (see Fig. 1). In these studies, observers typically maintain an “accessory” representation in WM (e.g., “red”) while concurrently using a different attentional template to search for an unrelated target among non-targets (e.g., a diamond among circles). On some trials, a salient non-target is presented in a color different from the others, which attracts visual attention and increases reaction times (RTs). Critically, visual search is disrupted more strongly on trials where the color of the salient non-target matches the “accessory” representation (e.g., a red circle). This memory-based interference was observed when search targets remained fixed through blocks of trials (Gunseli et al., 2016; Kim & Cho, 2016; Kumar et al., 2009; Olivers et al., 2006; Soto et al., 2005; Soto et al., 2008; van Moorselaar et al., 2014). In this situation, the corresponding attentional template may be transferred to LTM (Carlisle et al., 2011; Gunseli et al., 2014; Reinhart et al., 2014; Reinhart et al., 2016; Reinhart & Woodman, 2014, 2015; Woodman et al., 2013; Woodman et al., 2007), allowing the “accessory” representation to become “active” in WM and to interfere with the ongoing search task. In contrast, when the search target changes on a trial-by-trial basis, the corresponding attentional template is continuously updated in WM, allowing it to conserve its “active” status. Therefore, the other representation remains “accessory” and no memory-based interference occurs (Downing & Dodds, 2004; Hollingworth & Hwang, 2013; Houtkamp & Roelfsema, 2006; Olivers, 2009, Experiment 5; Peters et al., 2008; Woodman & Luck, 2007). While this theoretical framework (Olivers et al., 2011) received considerable support over the past years, a growing body of evidence challenges its core assumptions. First, studies using similar paradigms showed that more than one representation was able to interact with visual search at a time (Carlisle & Woodman, 2019; van Loon et al., 2017; Zhou et al., 2020). For instance, it has been demonstrated that memory-based interference increased with two “accessory” representations and two corresponding distractors (Chen & Du, 2017; Fan et al., 2019; Frătescu et al., 2019; Hollingworth & Beck, 2016). Second, memory-based interference has been reported in conditions where both the attentional template and the “accessory” representation were maintained in WM, which should not occur if the attentional template is the only “active” representation (Bahle et al., 2018; Foerster & Schneider, 2018; Kerzel & Andres, 2020; Zhang et al., 2018). Finally, as WM representations are stored in a distributed manner across sensory, parietal, and prefrontal networks (Christophel et al., 2017; D'Esposito & Postle, 2015; Postle, 2006), some argue that a bottleneck limiting attentional guidance to a single representation is unlikely, as that would require a singular, WM-specific neural mechanism (Kristjánsson & Kristjánsson, 2018).

Consistent with these observations, the multiple-template hypothesis (Beck et al., 2012) holds that several WM representations can guide visual attention simultaneously. That is, a small set of representations may be maintained in the “active” state and interact with visual search, which is in line with less restrictive state-based models of WM (Cowan, 1999, 2005; Gilchrist & Cowan, 2011; Oberauer & Bialkova, 2011). The major evidence in favor of this proposal stems from a second line of research that used dual-target search (see Fig. 1). In these tasks, observers employ one or two attentional templates (e.g., “red” or “red and blue”) to search for a target that is always defined by one of these target features (e.g., “red”). The idea is to compare single- with dual-target search with respect to overall performance or attentional capture. In this context, behavioral (Ansorge et al., 2005; Bahle et al., 2020; Huynh Cong & Kerzel, 2020; Irons et al., 2012; Kerzel & Witzel, 2019; Moore & Weissman, 2010; Roper & Vecera, 2012), electrophysiological (Berggren et al., 2020; Christie et al., 2014; Grubert & Eimer, 2015, 2016), and eye-movement (Beck & Hollingworth, 2017; Beck et al., 2012) studies showed that observers can concurrently employ two color templates. However, two simultaneous attentional templates might not be as efficient as a single attentional template. In fact, another body of studies reported performance impairments when observers searched for two possible targets relative to a single target, whether the relevant feature was shape (Houtkamp & Roelfsema, 2009), orientation (Barrett & Zobay, 2014), color (Dombrowe et al., 2011; Grubert et al., 2016; Stroud et al., 2011), or a combination of these three dimensions (Biderman et al., 2017). While the difference in search efficiency between one and two concurrently active attentional templates could reflect the switch from “accessory” to “active” state in WM (Ort et al., 2017; Ort et al., 2018; Ort & Olivers, 2020), the simultaneous guidance of visual search by two attentional templates confirms that more than one representation can be “active” in WM (Bahle et al., 2020).

## WM resources and visual search

Resource models of WM (Franconeri et al., 2013; Ma et al., 2014) may constitute powerful and parsimonious theoretical frameworks to give new insights on these issues. Particularly, differences in the allocation of resources between WM representations may have been neglected in previous studies (see Fig. 1). In dual-task paradigms, it is plausible to assume that more WM resources were allocated to the attentional template than to the “accessory” representation, which may account for absent or reduced memory-based interference. In contrast, there is no reason for unbalanced allocation of WM resources between two equally relevant attentional templates in dual-target search, even if fewer WM resources may be available with two than one attentional template. Based on these considerations, a few studies hypothesized that a representation may act as an attentional template depending on the amount of WM resources it receives (Dube & Al-Aidroos, 2019; Dube, Lumsden, et al., 2019b; Hollingworth & Hwang, 2013; Kerzel & Witzel, 2019). Specifically, the goal-relevance of the stimuli may determine the allocation of resources in WM, such that the most relevant ones are represented with a larger share of resources (Bays et al., 2011; Bays & Husain, 2008; Dube et al., 2017; Emrich et al., 2017; Gorgoraptis et al., 2011; Salahub et al., 2019; Zokaei et al., 2011). Allocating the largest amount of resources to a representation enhances its precision in WM and may allow it to guide visual search. In this section, we review empirical evidence from different lines of research about the value of this proposal. We proceed by answering the two following questions. Given that WM resources may play a role in visual search, is there a general relationship between the precision of attentional templates and visual search? Further, does the allocation of WM resources determine whether representations act as attentional templates?

### Is there a general relationship between attentional template precision and visual search?

The main evidence for a general relationship between the precision of attentional templates and visual search comes from studies using realistic objects as stimuli. Typically, these studies employed verbal or pictorial cues (e.g., the name of the object category or a picture) to specify the target’s features prior to visual search. While both types of cue allow setting up attentional templates, search is consistently less efficient with verbal than pictorial cues, as less visual information is available (Castelhano et al., 2008; Schmidt & Zelinsky, 2009; Vickery et al., 2005; Wolfe et al., 2004; Yang & Zelinsky, 2009). For instance, Schmidt and Zelinsky (2009) showed that the efficiency of visual search was directly related to the specificity of the cue. In different conditions, the cue was an exact picture of the target (e.g., “boots”), a precise textual description including color (e.g., “brown boots”), a precise textual description without color (e.g., “boots”), an abstract textual description including color (e.g., “brown footwear”), or an abstract textual description without color (e.g., “footwear”). Results showed that attentional guidance, indexed by fixation and saccade metrics, improved as more information was added to the attentional template. Confirming this observation with visual stimuli only, Hout and Goldinger (2015) used cues that represented the search target from a different viewpoint or that represented different exemplars from the same category. Compared with a condition where search targets exactly matched the previewed cues, both manipulations increased the number of saccades before the target was located, indicating that attentional guidance was impaired. Consistent with these results, it has been demonstrated that imprecise attentional templates resulted in inefficient search (Jenkins et al., 2018; Malcolm & Henderson, 2009, 2010; Nako, Wu, Smith, et al., 2014b), and that precise attentional templates improved visual search (Bravo & Farid, 2009, 2014; Castelhano et al., 2008; Nako, Wu, & Eimer, 2014a; Schmidt & Zelinsky, 2009, 2017; Vickery et al., 2005; Wolfe et al., 2004; Yang & Zelinsky, 2009). Taken together, these results demonstrate that adding details to the attentional template, and thus increasing its precision, directly enhances its efficiency in guiding attentional selection. However, this conclusion may be compromised by the very nature of the reviewed studies, that is, the use of realistic objects as stimuli. For instance, setting up attentional templates for realistic objects based on visual information may benefit from the reinstatement of object features from LTM (Kerzel & Andres, 2020), which may contribute to the advantage of pictorial compared with verbal cues. Moreover, it remains poorly understood whether all features of an object encoded in WM can interact with visual search. Some proposed that object features are processed individually (Olivers et al., 2006; Sala & Courtney, 2009), whereas others argued that it is an object-based phenomenon (Foerster & Schneider, 2018; Gao et al., 2016; Soto & Humphreys, 2009). Finally, the definition of precision differs considerably in the reviewed studies compared with the WM literature and may not reflect the same underlying mechanism. In the comparison of verbal and pictorial cues, precision is conceptualized as the number of features available to specify a single visual object. In research on WM resources, precision refers to the width of the response distribution for several individual features recalled from WM (see below). So far, only the latter approach has been used to quantify the continuous allocation of resources in WM, which is necessary to conclude on the relationship between the precision of attentional templates and visual search. In addition, the fine assessment of resource allocation allows distinguishing between two causal directions. Possibly, the allocation of the largest amount of resources to a WM representation grants this representation the status of attentional template. Alternatively, obtaining the status of attentional template results in the allocation of the largest amount of resources to the corresponding representation.

### Does the allocation of WM resources determine whether representations act as attentional templates?

**Fig. 2:**
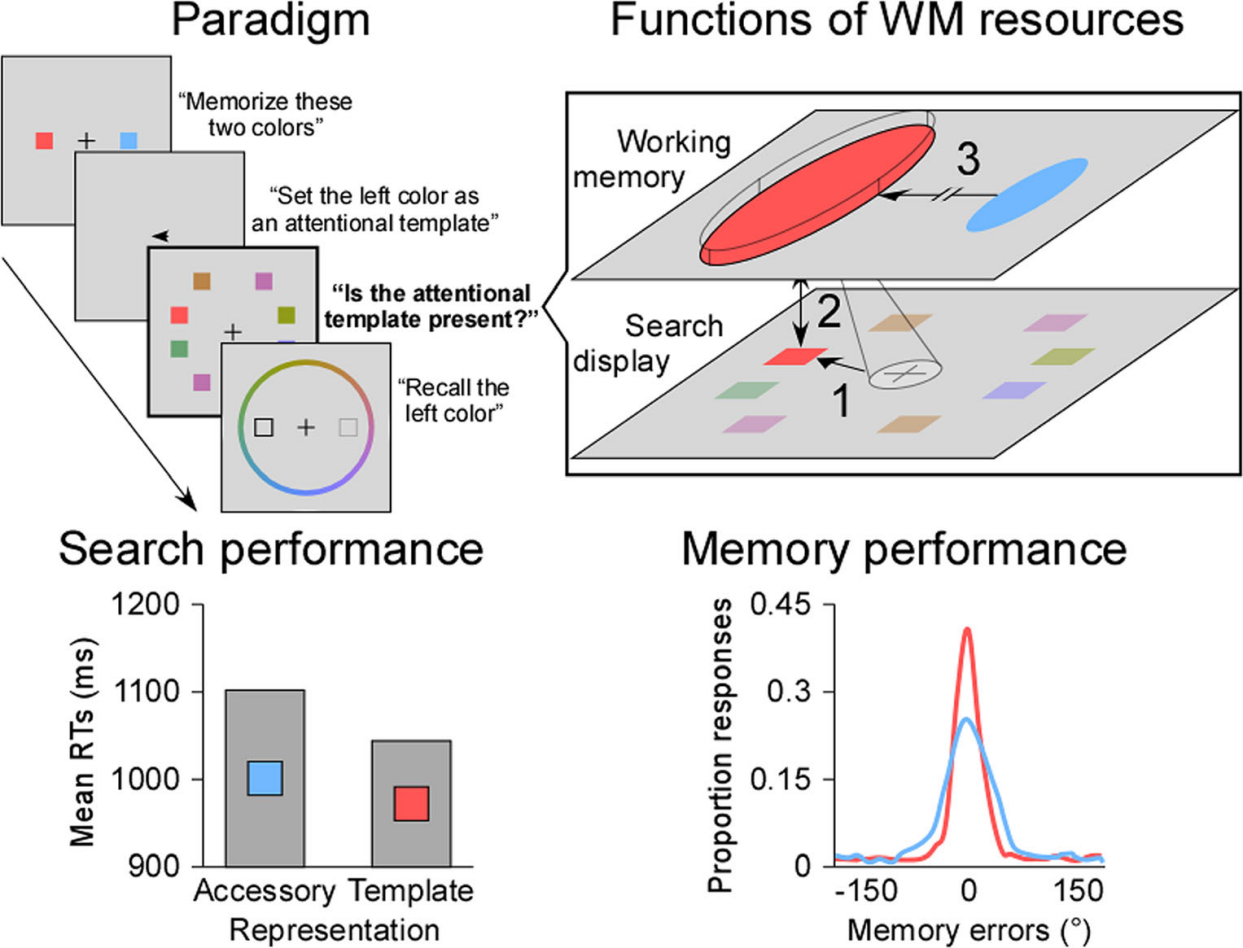
Experimental procedure of visual search combined with a continuous delayed-estimation task, typical results, and the three hypothetical functions of working memory (WM) resources in visual search. In an example trial inspired by Rajsic and Woodman (2019), observers were asked to memorize two colors and to set one as an attentional template by retro-cueing (upper left panel). Then, observers had to indicate whether the color of the attentional template was present or absent in the search display and to recall one of the two memorized colors. Similar to a condition where neither color was present, mean reaction times (RTs) were delayed when the search display contained the “accessory” color compared with the attentional template (data from their Experiment 1, lower left panel). Moreover, analysis of the memory performance showed that the attentional template was always recalled more precisely than the “accessory” color (lower right panel). In this situation, the resource hypothesis of visual search states that the attentional template received the largest amount of resources in WM (upper right panel), which improved attentional guidance by increasing the selection bias in favor of relevant features (arrow 1), facilitated target recognition by accelerating the match with potential targets (arrow 2), and/or protected the attentional template from the interference caused by the “accessory” color in WM (arrow 3)

As introduced earlier, resources are assumed to reduce the noise in internal representations of sensory stimuli, which enhances their precision of recall (Ma et al., 2014). On this basis, a few studies assessed the amount of WM resources allocated to attentional templates by measuring their recall precision in continuous delayed-estimation tasks (see Fig. 2). Typically, observers are asked to encode two stimuli in WM whose respective relevance is determined by explicit task instructions or is manipulated afterwards with *retro-cues* (Landman et al., 2003; Nobre et al., 2008; Souza & Oberauer, 2016). Then, after having performed an intervening visual search, observers reproduce one of the memorized stimuli using a continuous scale (e.g., choosing a color on a color wheel). Compared with traditional change detection procedures, this recall technique allows for the precise measurement of the distance between the true and the judged feature on each trial, whether it is color, orientation, or motion direction (Fougnie et al., 2012; Gorgoraptis et al., 2011; Rademaker et al., 2012; Wilken & Ma, 2004; Zokaei et al., 2011). In doing so, it is possible to submit the distribution of memory errors to modeling and to identify different sources of error. While a number of models have been proposed to decompose such data (e.g., Luck & Vogel, 1997; Oberauer & Lin, 2017; van den Berg et al., 2012; Zhang & Luck, 2008), the three-parameter mixture model of Bays et al. (2009) has been most commonly applied to template-guided visual search (Dube & Al-Aidroos, 2019; Hollingworth & Hwang, 2013; Huynh Cong & Kerzel, 2020; Kerzel, 2019; Kerzel & Witzel, 2019; Rajsic et al., 2017; Rajsic & Woodman, 2019). In this model, three distributions contribute to the likelihood of a given response. Namely, a uniform distribution that reflects the proportion of random guesses (P_Guess_), a von Mises distribution that reflects the precision of responses to the probed item (P_SD_), and a von Mises distribution that reflects the proportion of responses to the non-probed item (P_Swap_). In the theoretical interpretation of these parameters, only P_SD_ is assumed to reflect the continuous allocation of WM resources to the corresponding representations (for an extended discussion of these parameters, see Ma et al., 2014). However, it is worth noting that the most recently proposed continuous-resource models, termed variable-precision models (Fougnie et al., 2012; van den Berg et al., 2014; van den Berg et al., 2012), have not yet been employed in the context of template-guided visual search.

Hollingworth and Hwang (2013) were the first to investigate whether representations receiving the largest amount of WM resources acted as attentional templates. Their initial answer was negative, but the following discussion gave way to a more differentiated view. Hollingworth and Hwang (2013) presented two colors to memorize, followed by an 80% valid retro-cue indicating which color would be probed more often. Both memory-based interference in a search task and recall precision were evaluated. Search times were not prolonged by distractors matching the non-cued color compared with distractors matching an unrelated color, suggesting that the non-cued representation did not specifically impair visual search. However, the non-cued color was recalled with the same precision as the cued color, indicating that the respective representations received an equal amount of resources in WM. Because the representation of the non-cued color did not act as an attentional template despite its equal share of resources, it was concluded that memory precision does not determine the status of WM representations for visual search. However, as memory-based interference from distractors matching the cued color was not measured, the possibility remains that neither cued nor non-cued representations were able to interact with visual search. Subsequent studies adjusted the procedure used by Hollingworth and Hwang (2013) and found higher recall precision for cued than non-cued colors, replicating this time the expected retro-cueing effect (Souza & Oberauer, 2016). Further, search times were longer with a distractor matching the cued than the non-cued colors when the retro-cue was 100% valid, but not when it was 80% or 70% valid (Dube & Al-Aidroos, 2019; Dube, Lumsden, et al., 2019b). As expected, allocating the largest amount of resources to a representation increased its precision in WM. However, receiving the largest amount of resources was not sufficient for this representation to act as an attentional template. The only exception are 100% valid retro-cues, which allowed the corresponding representations to interact with visual search (Dube, Lumsden, et al., 2019b). In this situation, the amount of resources allocated to the cued representation may have exceeded a threshold that was not reached with 70% or 80% valid retro-cues. That is, only with sufficient resources do WM representations access the status of attentional template. However, the cued representation was recalled with similar precision with 70% and 100% valid retro-cues (Dube, Lumsden, et al., 2019b), indicating that the allocation of WM resources was not different in these two conditions. Moreover, previous studies repeatedly showed that a single representation maintained in WM, which is supposed to receive all available resources, did not necessarily act as an attentional template (e.g., Carlisle & Woodman, 2011; Downing & Dodds, 2004; Houtkamp & Roelfsema, 2006; Woodman & Luck, 2007). Therefore, it seems unlikely that the allocation of resources plays a critical role in determining whether WM representations can interact with visual search or not. Instead, an additional process may be responsible for granting the status of attentional template to WM representations (see *Theoretical implications*).

While not *sufficient*, the allocation of the largest amount of resources may be *necessary* for WM representations to act as attentional templates. Using single- and dual-target search, studies provided convincing evidence in favor of this assumption. For instance, Rajsic et al. (2017) asked observers to maintain two representations for subsequent recall. Rather than using a retro-cue to indicate which representation would be probed more often, they used a retro-cue to indicate which one would serve as the attentional template for the intervening search task (see Fig. 2). Results showed that assigning the status of attentional template to a representation in WM increased the probability and precision of its recall, regardless of the occurrence of search (Rajsic et al., 2017) and its difficulty (Rajsic & Woodman, 2019). Thus, following the balanced allocation of WM resources between two representations, the subsequent attribution of the attentional template status induced a reallocation of WM resources in favor of the corresponding representation. Consistent with these observations, Kerzel and Witzel (2019) showed that directly encoding a color as an attentional template also led to the allocation of the largest amount of WM resources. Interestingly, however, the subsequent reallocation of WM resources away from this attentional template was not under voluntary control. In Kerzel and Witzel (2019), observers were asked to memorize the target and distractor colors for visual search and subsequent recall. To evaluate whether an attentional template had been set up for each of these colors, the contingent capture paradigm (Folk & Remington, 1998; Folk et al., 1992) was used. Cueing effects were observed for the target color but not for the distractor color, indicating that an attentional template had been set up for the target, but not for the distractor. At the same time, the recall precision of the distractor color was consistently worse than the recall precision of the target color although observers were instructed to recall the distractor color with equal or better precision than the target color. Thus, WM resources allocated to the attentional template could not be reallocated to another representation despite instructions to do so and frequent feedback. Taken together, these results indicate that the allocation of the largest amount of WM resources to an attentional template seems to be an unavoidable consequence of becoming an attentional template and cannot be easily reversed thereafter. While this may be true when a single attentional template is concurrently maintained with another WM representation (Kerzel & Witzel, 2019; Rajsic et al., 2017; Rajsic & Woodman, 2019), the allocation of resources between two or more attentional templates may be balanced and flexibly adjusted. Consistently, the only study that investigated this question (Huynh Cong & Kerzel, 2020) suggests that WM resources are allocated and reallocated between two attentional templates depending on their respective relevance for the task at hand (see *Protection from interference*). Therefore, it would be more appropriate to conclude that representations obtaining the status of attentional template receive an amount of WM resources proportional to their relevance for visual search. In other words, a single attentional template receives the largest amount of WM resources since it is the only relevant representation for visual search, whereas multiple attentional templates receive an amount of WM resources that depends on their relevance for the search task. In any case, by directly assessing the allocation of resources in WM, this line of research provides converging evidence that the precision of attentional templates may have a functional value in visual search (Bravo & Farid, 2009, 2014; Castelhano et al., 2008; Jenkins et al., 2018; Malcolm & Henderson, 2009, 2010; Nako, Wu, & Eimer, 2014a; Nako, Wu, Smith, et al., 2014b; Schmidt & Zelinsky, 2009, 2017; Vickery et al., 2005; Wolfe et al., 2004; Yang & Zelinsky, 2009).

### Theoretical implications

Based on resource models of WM (Franconeri et al., 2013; Ma et al., 2014), a few studies hypothesized that the amount of WM resources allocated to a representation enhances its recall precision and may determine its ability to guide visual search (Dube & Al-Aidroos, 2019; Dube, Lumsden, et al., 2019b; Hollingworth & Hwang, 2013; Kerzel & Witzel, 2019). While this simple connection between WM resources and template-guided visual search seems appealing, the evidence examined earlier shows that it is empirically untenable. Instead, we propose an extensive and comprehensive framework based on resource models of WM and the literature that has been extensively reviewed above. The *resource hypothesis of visual search* comprises a set of three main principles to conceptualize the complex relationships between WM resources and attentional templates. Here, we expose each of the three principles that constitute this hypothesis and discuss their relevance in relation to existing models of template-guided visual search.

First, the allocation of resources to an attentional template has an effect on visual search. While appearing trivial, this first principle received considerable amount of support from studies that manipulated the number of features specifying attentional templates and demonstrated clear causal effects on visual search (Bravo & Farid, 2009, 2014; Castelhano et al., 2008; Jenkins et al., 2018; Malcolm & Henderson, 2009, 2010; Nako, Wu, & Eimer, 2014a; Nako, Wu, Smith, et al., 2014b; Schmidt & Zelinsky, 2009, 2017; Vickery et al., 2005; Wolfe et al., 2004; Yang & Zelinsky, 2009). Consistently, studies that directly assessed the allocation of WM resources during visual search observed the highest recall precision for attentional templates compared with other representations (Kerzel & Witzel, 2019; Rajsic et al., 2017; Rajsic & Woodman, 2019). Although it remains unclear whether these two lines of research describe the same underlying mechanism, they provide converging evidence that the precision of attentional templates, and presumably the allocation of WM resources, affects visual search. Therefore, these findings are critical in extending resource models of WM to template-guided visual search. In addition to increasing recall precision of stored representations, WM resources may serve additional functions in visual search such as enhancement of attentional guidance, facilitation of target recognition, and/or protection against interference. We address each of these hypotheses in the following section.

Second, as laid out above, the allocation of the largest amount of resources to a representation in WM is not sufficient to give this representation the status of attentional template and thus, the ability to guide visual search (Dube & Al-Aidroos, 2019; Dube, Lumsden, et al., 2019b; Hollingworth & Hwang, 2013). That is, the allocation of resources in WM is unlikely to determine the status of a representation for visual search. In these terms, the resource hypothesis of visual search is compatible with two proposals that attribute the status of attentional template to other processes, such as goal-dependent executive control and less restricted “active” states in WM. According to the first of these accounts, executive control may trigger a biasing signal before WM representations can interact with visual search, thus mediating the relation between WM and visual search (Bundesen et al., 2005). In that sense, WM representations would act as attentional templates only when goal-relevant in the search task (Carlisle & Woodman, 2011; Downing & Dodds, 2004; Peters et al., 2008; Woodman & Luck, 2007). As a prime example in favor of this proposal, Woodman and Luck (2007) observed the presence of memory-based interference only when observers knew that the “accessory” representation could be the search target on some trials, but not when it was never the search target. Since this manipulation of probability affected the interaction of WM representations and visual search, it was proposed that the status of attentional template may depend on higher-level strategies that relate to executive control. As an alternative to this account, and closely related to the dual-state model (Olivers et al., 2011), the attentional template status may be determined by an “active” representational state in WM granted by the focus of attention. However, instead of being restricted to a single representation (McElree, 2001, 2006; Oberauer, 2002, 2009; Oberauer & Hein, 2012), the focus of attention may be broader, thus comprising multiple “active” representations in WM (Cowan, 1999, 2005; Gilchrist & Cowan, 2011; Oberauer & Bialkova, 2011). Therefore, contrary to the initial dual-state model of Olivers et al. (2011), more than one WM representation would be able to act as an attentional template (Bahle et al., 2020). While these two views describe the processes that determine the status of attentional template, they both need to include an additional mechanism to account for differences between multiple “goal-relevant” or “active” representations. That is, once WM representations are set up as attentional templates by executive control or the broad focus of attention, resources may be flexibly allocated between them as a function of their relevance for the task at hand. Consistent with this idea, Bahle et al. (2020) noted that “it is plausible that, even if multiple items are maintained in a state that interacts with attention, there will be differences in their absolute levels of activity (or priority)” (p. 2). Therefore, the resource hypothesis of visual search may be an extension to existing proposals by accounting for situations where multiple attentional templates are simultaneously required.

Third, representations that obtain the status of attentional template, whether at encoding or during maintenance, receive an amount of WM resources proportional to their relevance for visual search. Therefore, a single attentional template receives the largest amount of WM resources because it is the only relevant representation (Kerzel & Witzel, 2019; Rajsic et al., 2017; Rajsic & Woodman, 2019), whereas two or more attentional templates receive an amount of WM resources that depends on their respective relevance (Huynh Cong & Kerzel, 2020). Interestingly, however, these studies also suggest that the reallocation of WM resources between an attentional template and another representation may not be as flexible as between multiple attentional templates. While WM resources can be reallocated between two attentional templates on a trial-by-trial basis (Huynh Cong & Kerzel, 2020) and toward one of two representations that will act as an attentional template (Rajsic et al., 2017; Rajsic & Woodman, 2019), WM resources cannot be reallocated from the attentional template to another representation (Kerzel & Witzel, 2019). These observations corroborate the idea that attentional templates possess a different status in WM compared with search-unrelated representations (Carlisle & Woodman, 2011; Olivers & Eimer, 2011), which may constrain the reallocation of resources between these two types of representations. As discussed above, this assumption is perfectly in line with models of template-guided visual search proposing that attentional templates are “active” (e.g., Bahle et al., 2020) or “goal-relevant” (e.g., Woodman et al., 2007) representations in WM. However, these observations further suggest that differences in status may be associated with differences in how flexibly WM processes, such as the reallocation of resources, can operate on these representations. Finally, it is worth noting that the difficulty in reallocating resources from an attentional template to another representation (Kerzel & Witzel, 2019) is also consistent with recent proposals that the initial allocation of WM resources is automatically driven, while the subsequent reallocation of resources depends on controlled processes that are considerably limited (Dube, Lockhart, et al., 2019a; Williams et al., 2020). Thus, representations that obtain the attentional template status may automatically bias the allocation of WM resources in their favor, with little possibility for controlled processes to reverse this situation. However, further investigations are required to address at least two issues regarding this assumption. First, attentional templates were always goal-relevant in the reviewed studies (Huynh Cong & Kerzel, 2020; Kerzel & Witzel, 2019; Rajsic et al., 2017; Rajsic & Woodman, 2019), making it impossible to conclude on the presence of an automatic process. That is, the initial allocation of WM resources toward attentional templates corresponded to task requirements so that automatic and controlled processes could not be dissociated. Second, and importantly, this proposal does not provide a clear explanation for why controlled processes would be limited in reallocating WM resources between an attentional template and another representation (Kerzel & Witzel, 2019), but not between multiple attentional templates (Huynh Cong & Kerzel, 2020).

## Functions of WM resources in visual search

So far, we have shown that WM representations receiving the largest amount of resources do not necessarily act as attentional templates. However, single attentional templates inevitably receive the largest amount of resources, which makes them more precise than any other representations in WM. While the increase in resources appears to improve visual search, the exact processes involved are still to be determined. Here, we present three proposals about the role of WM resources in template-guided visual search that may not be mutually exclusive (see Fig. 2). For ease of exposition, the attentional guidance hypothesis and the target recognition hypothesis are discussed together. In contrast, the protection hypothesis is addressed separately since it specifies an additional function of WM resources that may be relevant only when interference occurs during visual search.

### Attentional guidance and target recognition

As introduced earlier, attentional templates contribute to two distinct processes in visual search. First, attentional templates allow for the selection of objects with template-matching attributes by converting display-wide enhancement of relevant features into spatially specific enhancement, thus guiding visual attention (Eimer, 2014; Moran & Desimone, 1985; Motter, 1994). Second, attentional templates allow for decisions about whether selected stimuli match the target (Cunningham & Wolfe, 2014) until search is successful or a termination criterion is met (Wolfe & Van Wert, 2010). Thus, the precision of attentional templates may improve visual search by enhancing attentional guidance, by facilitating recognition and decision processes, or both. Concerning attentional guidance, more precise attentional templates may increase the selection bias in favor of relevant features and guide visual attention to fewer potential targets during search. That is, the amount of WM resources allocated to an attentional template should be directly linked to its search efficiency. For instance, event-related potential (ERP) studies demonstrated that the precision of the attentional template had a direct effect on the N2pc component (Jenkins et al., 2018; Nako, Wu, & Eimer, 2014a; Nako, Wu, Smith, et al., 2014b), known to index attentional selection of objects with template-matching features at relatively early stages of visual processing (Eimer, 1996; Eimer & Kiss, 2008; Leblanc et al., 2007; Lien et al., 2008; Luck & Hillyard, 1994). Concerning target recognition, more precise attentional templates may accelerate the match with potential targets, once they have been localized. Thus, the time needed to recognize the target and make a decision should depend on the amount of WM resources received by the attentional template. While RTs are consistent with both accounts (e.g., Kerzel & Witzel, 2019; Rajsic et al., 2017), eye-tracking studies were able to precisely measure the effect of attentional templates on these two stages of visual search. For instance, Castelhano et al. (2008) showed that precise attentional templates improved visual search by shortening the verification time, that is, the time needed to respond to the search target once it was fixated. Subsequent studies replicated the effect of precision on verification time and additionally found that more precise attentional templates reduced the scan time, indicating that attentional template precision affected both the guidance of visual attention and target recognition (Hout & Goldinger, 2015; Malcolm & Henderson, 2009, 2010; Schmidt & Zelinsky, 2009). Taken together, these studies suggest that the precision of attentional templates, as defined by the number of specifying features, may influence both search processes rather than only one. As mentioned previously, however, this definition of precision may not exactly reflect the continuous allocation of resources in WM. Therefore, converging evidence from direct measures of recall precision is needed. In that sense, Rajsic and Woodman (2019) recently demonstrated that the allocation of WM resources was more likely to serve recognition and decision instead of attentional guidance. The rationale of their study was the following. If attentional templates are represented more precisely in WM to improve search efficiency, observers should strategically increase the amount of resources dedicated to an attentional template when visual search is difficult relative to when visual search is easy. The reason is that the target may be detected pre-attentively in easy visual search where the target pops out in the search display (Bacon & Egeth, 1994; Treisman & Gelade, 1980). Thus, increasing resources in easy visual search would not improve attentional guidance any further, but it would do so in difficult visual search. In contrast, if attentional template precision is important to decide about the presence of the target, the amount of WM resources dedicated to an attentional template should be similar in difficult and easy visual search. The reason is that simply preparing a representation for comparison with incoming visual input is sufficient to induce memory benefits for it or costs for other representations (Myers et al., 2017; Reinhart & Woodman, 2014; Souza et al., 2015; Zokaei et al., 2014), irrespective of the search difficulty. Results showed that attentional templates were always recalled more precisely than other WM representations, regardless of whether visual search was difficult or easy (but see Schmidt & Zelinsky, 2017). Consistent with studies that manipulated the number of features specifying attentional templates (e.g., Castelhano et al., 2008), these observations support the target recognition hypothesis. However, it appears premature to conclude that WM resources affect only one stage of visual search because so far only Rajsic and Woodman (2019) have directly measured the recall precision of attentional templates in this context. Further investigations are necessary to determine under which circumstances the precision of an attentional template, as measured with continuous delayed-estimation tasks, improves the guidance of visual attention, facilitates target recognition, or both.

### Protection from interference

Recently, Berggren et al. (2020) investigated dual-target search that involved the simultaneous activation of a transient template in WM and a template held in a sustained fashion in LTM. That is, one of the two target colors varied on a trial-by-trial basis whereas the other remained fixed throughout. Surprisingly, search performance was worse for the fixed than the variable target color, which suggests that the encoding of the transient template in WM retroactively interfered with the maintenance of the sustained template in LTM. Although the distinction between sustained and transient templates is assumed to reflect a strict dichotomy between WM and LTM (Carlisle et al., 2011; Woodman et al., 2013), LTM representations may be retrieved and buffered within WM to be accessed consciously and to affect online task performance (Cantor & Engle, 1993; Cowan et al., 2013; Fukuda & Woodman, 2017; Nairne & Neath, 2001). Thus, sustained templates may be subject to characteristics associated with maintaining and processing information in WM, such as resource allocation. Based on this assumption, Huynh Cong and Kerzel (2020) hypothesized that the costs associated with the sustained template could simply reflect that more WM resources were allocated to the transient template. Following a dual-target search similar to Berggren et al. (2020)’s, observers were asked to recall either the sustained or transient template on a continuous scale. In addition to replicating the RT costs, Huynh Cong and Kerzel (2020) showed that the sustained template was more often forgotten when paired with a transient template, indicating that retroactive interference affected visual search and memory maintenance alike. However, when the sustained template was not forgotten, its recall precision was highest, but its search efficiency was still considerably impaired. This specific pattern of results is incompatible with the attentional guidance and target recognition hypotheses as more precise attentional templates were expected to improve visual search, which was not the case (see also Kerzel, 2019). Given this inconsistency, an additional hypothesis about the function of WM resources in visual search must be considered. Particularly, WM resources may serve to protect an attentional template when there is interference from competing attentional templates, rather than to improve visual search. Consistent with this idea, Huynh Cong and Kerzel (2020) found that balancing WM resources between sustained and transient templates reduced interference and that allocating the largest amount of resources to the sustained template made interference disappear. Therefore, the protection hypothesis may explain seemingly paradoxical situations where memory performance is good, but the respective WM representation acts poorly (or not at all) as an attentional template. That is, protection by the allocation of WM resources may allow for a precise representation of the attentional template despite interference, but does not guarantee its ability to efficiently guide visual search. While the protective effect of WM resources is most clearly illustrated by retroactive interference between two concurrently active attentional templates, it may apply to other conditions as well. For instance, previous results showing that WM representations were recalled with high precision, but did not always interact with visual search (Dube & Al-Aidroos, 2019; Dube, Lumsden, et al., 2019b; Hollingworth & Hwang, 2013), may also reflect that resources served to protect these representations from mutual interference in WM. However, the exact nature and conditions of interference that necessitate protection are to be determined. In its current form, the protection hypothesis is a new assumption that could only be formulated after assessing the allocation of WM resources between two concurrently active attentional templates, which has been rarely done. Indeed, previous studies mainly focused on the allocation of resources between an attentional template and another WM representation (Kerzel & Witzel, 2019; Rajsic et al., 2017; Rajsic & Woodman, 2019) or between two WM representations maintained during visual search (Dube & Al-Aidroos, 2019; Dube, Lumsden, et al., 2019b; Hollingworth & Hwang, 2013). However, determining the functional value of WM resources in dual-target search is critical. From a theoretical standpoint, it is necessary to elaborate how multiple “goal-relevant” or “active” representations interact (Bahle et al., 2020) and compete with each other in WM (Oberauer et al., 2012; Oberauer & Lin, 2017) for the guidance of visual search.

While no previous study investigated protection from interference in the context of template-guided visual search, this topic has been particularly fruitful in the WM literature. First, studies investigating individual differences have repeatedly demonstrated that interference impaired memory performance in individuals with low WM capacity, but not in those with high WM capacity (Engle, 2002; Kane et al., 2001; Kane & Engle, 2000; Rosen & Engle, 1997). As interference slows and impairs memory retrieval, maintaining goal-relevant information highly active and easily accessible requires more resources than if interference was absent. That is, only individuals allocating more resources to goal-relevant representations would be able to actively maintain them and to protect them from interference. Second, numerous studies that employed retro-cues to manipulate the relevance of stimuli, and thus the allocation of resources in WM, reported strengthening and protective effects (Souza & Oberauer, 2016). Similar to refreshing, retro-cues make the corresponding representations, and the binding to their context (e.g., their spatial location), stronger than they were right after encoding, which improves the accessibility for later use (Kuo et al., 2011; Lepsien et al., 2011; Nobre et al., 2008; Rerko & Oberauer, 2013; Rerko et al., 2014; Souza et al., 2015; Vandenbroucke et al., 2011). Moreover, these representations are also protected from interference by visual inputs during the retention interval or at recall, whereas unprotected representations are impaired (Makovski & Jiang, 2007; Matsukura et al., 2007; Sligte et al., 2008; Souza et al., 2016). Therefore, retro-cues increase the precision of representations in WM and allow these representations to conserve their precision in the face of interference. Taken together, these results pave the way for the assumption that WM resources could also serve a protective function in template-guided visual search.

## Open questions

Behavioral evidence from continuous delayed-estimation tasks indicates that single attentional templates receive the largest amount of WM resources as their recall precision is higher than the recall precision of other WM representations. However, these measures are usually collected after visual search is performed, allowing intervening stimuli to contaminate them. For instance, orienting visual attention toward a distractor disrupts information already stored in WM (Hamblin-Frohman & Becker, 2019; Tas et al., 2016; Williams et al., 2020), orienting visual attention to the search target improves its precision in memory (Huynh Cong & Kerzel, 2020; Kerzel & Witzel, 2019; Maxcey-Richard & Hollingworth, 2013; Rajsic & Woodman, 2019; Woodman & Luck, 2007), and adding details to the probing scale can interfere with the retrieval of WM representations (Souza et al., 2016; Tabi et al., 2019). Moreover, recall precision is a parameter that depends on the model used to decompose memory errors with considerable differences between their estimates (Bays et al., 2009; Luck & Vogel, 1997; Oberauer & Lin, 2017; van den Berg et al., 2014; van den Berg et al., 2012; Zhang & Luck, 2008). For these reasons, electrophysiological investigations of resource allocation in WM may be an interesting avenue for future research. In fact, recent ERP studies showed that the CDA (Luria et al., 2016; Vogel & Machizawa, 2004; Vogel et al., 2005) may track the active maintenance of attentional templates in WM (Woodman & Arita, 2011), the transfer of attentional templates from WM to LTM (Carlisle et al., 2011; Woodman et al., 2013), the importance given to search performance in an upcoming trial (Reinhart et al., 2016; Reinhart & Woodman, 2014), and the selective encoding of attentional templates (Rajsic et al., 2020). Further, the amplitude of the CDA has also been linked to the precision of representation in WM (Luria et al., 2009; Machizawa et al., 2012; Schmidt & Zelinsky, 2017) and the flexible allocation of WM resources (Salahub et al., 2019). In a similar vein, posterior alpha-band oscillations have proven useful in investigating the attentional prioritization and suppression of representations within WM (de Vries et al., 2020; de Vries et al., 2018; de Vries et al., 2017, 2019; Myers et al., 2015; Poch et al., 2018; Schneider et al., 2019; Schneider et al., 2015, 2016; van Ede, 2018). Systematically using these electrophysiological techniques would allow future research to better understand the interactions between WM resources and template-guided visual search. In that sense, Table 2 presents a non-exhaustive list of open questions that should be addressed with priority to develop the resource hypothesis of visual search.

**Table 2:** Open questions

Topic	Question
Allocation and reallocation of WM resources toward attentional templates and search-unrelated representations	To what extent do the allocation and reallocation of resources depend on automatic and controlled processes for attentional templates? Particularly, does it involve an automatic allocation, followed by a controlled reallocation as suggested by Williams et al. (2020)? Moreover, can the reallocation of WM resources only occur when it is goal-relevant? And, is the reallocation of WM resources more flexible between multiple attentional templates than between an attentional template and a search-unrelated representation?
Functions of WM resources in visual search	Under which circumstances does the allocation of WM resources improve attentional guidance, facilitate target recognition, or both?
Protection hypothesis	What are the conditions in which protection from interference is required for attentional templates? Does it only occur with multiple attentional templates? What are the types of interference (e.g., proactive, retroactive) that interact with the allocation of WM resources? Are there differential effects for template-guided visual search? Do individual differences in WM capacity modulate interference in template-guided visual search as suggested by Kane et al. (2001)?
Electrophysiological measures	Are the electrophysiological measures of the allocation of WM resources more reliable than recall precision? For instance, when intervening stimuli interfere during maintenance or contaminate the recall?

## Conclusions

Recently, WM has been conceptualized as a limited resource, distributed flexibly and strategically between stored representations. As attentional templates are thought to be represented in WM, we reviewed empirical evidence that the allocation of WM resources has consequences not only on memory, but also on visual search. We have argued that three main principles govern the relationships between WM resources and template-guided visual search. First, the allocation of resources to an attentional template has an effect on visual search, as it may improve the guidance of visual attention, facilitate target recognition, and/or protect attentional templates against interference. Second, the allocation of the largest amount of resources to a representation in WM is not sufficient to give this representation the status of attentional template and thus, the ability to guide visual search. Third, the representation that obtains the status of attentional template, whether at encoding or during maintenance, receives an amount of WM resources proportional to its relevance for visual search. Thus formalized, the resource hypothesis of visual search describes how the internal representation of the target is maintained in WM and how it affects the exploration of visual environments. Moreover, the concept of WM resources gives new insights on previous debates and complements existing models of template-guided visual search.

## Footnotes

1 Note that interpretation of these results remains an active area of debate since discrete-capacity models can be modified to behave like resource models. For instance, more than one slot may be dedicated to represent a stimulus, which would enhance its recall precision similar to an increase in resources (see Zhang & Luck, 2008).
